# Ultra-high-resolution 40 keV virtual monoenergetic imaging using spectral photon-counting CT in high-risk patients for coronary stenoses

**DOI:** 10.1007/s00330-024-11237-x

**Published:** 2024-12-11

**Authors:** Guillaume Fahrni, Sara Boccalini, Hugo Lacombe, Fabien de Oliveira, Angèle Houmeau, Florie Francart, Marjorie Villien, David C. Rotzinger, Antoine Robert, Philippe Douek, Salim A. Si-Mohamed

**Affiliations:** 1 Department of Diagnostic and Interventional Radiology, Cardiothoracic and Vascular Division, Lausanne University Hospital and University of Lausanne, Lausanne, Switzerland; 2 University of Lyon, INSA-Lyon, Université Claude Bernard Lyon 1, UJM-Saint Etienne, CNRS, Inserm, CREATIS, Villeurbanne, France; 3 Department of Cardiovascular and Thoracic Radiology, Louis Pradel Hospital, Hospices Civils de Lyon, Bron, France; 4 Philips Healthcare, Suresnes, France; 5 Department of Radiology, CHU Nîmes, University Montpellier, Medical Imaging Group Nîmes, Nîmes, France

**Keywords:** Multidetector computed tomography, Image enhancement, Comparative study, Diagnosis, Coronary arteries

## Abstract

**Objectives:**

To assess the image quality of ultra-high-resolution (UHR) virtual monoenergetic images (VMIs) at 40 keV compared to 70 keV, using spectral photon-counting CT (SPCCT) and dual-layer dual-energy CT (DECT) for coronary computed tomography angiography (CCTA).

**Methods and materials:**

In this prospective IRB-approved study, 26 high-risk patients were included. CCTA was performed both with an SPCCT in UHR mode and with one of two DECT scanners (iQOn or CT7500) within 3 days. 40 keV and 70 keV VMIs were reconstructed for both modalities. Stenoses, blooming artefacts, and image quality were compared between all four reconstructions.

**Results:**

Twenty-six patients (4 women [15%]) and 28 coronary stenoses (mean stenosis of 56% ± 16%) were included. 40 keV SPCCT gave an overall higher quality score (5 [5, 5]) than 70 keV SPCCT (5 [4, 5], 40 keV DECT (4 [3, 4]) and 70 keV SPCCT (4 [4, 5]), *p* < 0.001). Less variability in stenosis measurement was found with SPCCT between 40 keV and 70 keV (bias: −1% ± 3%, LoA: 6%) compared with DECT (−6% ± 8%, LoA 16%). 40 keV SPCCT vs 40 keV DECT showed a −3% ± 6% bias, whereas 40 keV SPCCT vs 70 keV DECT showed a −8% ± 6% bias. From 70 keV to 40 keV, blooming artefacts did not increase with SPCCT (mean +2% ± 5%, *p* = 0.136) whereas they increased with DECT (mean +7% ± 6%, *p* = 0.005).

**Conclusion:**

UHR 40 keV SPCCT VMIs outperformed 40 keV and 70 keV DECT VMIs for assessing coronary artery stenoses, with no impairment compared to 70 keV SPCCT VMIs.

**Key Points:**

***Question*** *Use of low virtual mono-energetic images at 40* *keV using spectral dual-energy and photon-counting CT systems is not yet established for diagnosing coronary artery stenosis*.

***Findings*** *UHR 40* *keV SPCCT enhances diagnostic accuracy in coronary artery assessment*.

***Clinical relevance*** *By combining spectral sensitivity with lower virtual mono-energetic imaging and ultra-high spatial resolution, SPCCT enhances coronary artery assessment, potentially leading to more accurate diagnoses and better patient outcomes in cardiovascular imaging*.

## Introduction

Coronary computed tomography angiography (CCTA) is increasingly used and recommended as a non-invasive test for coronary artery disease (CAD) assessment, especially in low and intermediate-risk patients, for both acute and chronic chest pain [1, 2]. Its diagnostic performance, closely linked to technical factors, has greatly increased with the recent progress in computed tomography (CT) technology [3]. Spectral dual-energy computed tomography (DECT) is the state-of-the-art technique for this clinical setting, achieving greater diagnostic performance for CAD with lower radiation doses than with conventional single-energy CT scanning [4]. Another advantage of DECT is the possibility to reconstruct virtual monoenergetic images (VMIs), serving as a surrogate for 120 kVp polychromatic images such as 70 kilo-electronvolt (keV) [5, 6], greatly improving contrast in low energy ranges such as 40 keV [7], and reducing blooming artefacts in high energy ranges such as 120 keV [8]. Furthermore, the use of low keV VMI has the potential to reduce iodine volume, which is of great interest considering the growing ecological concerns about iodine contamination in water, as well as the risk of venous complications for the patient [9, 10]. Nevertheless, DECT is still subject to several limitations, such as the use of energy-integrating detectors (EIDs), the same type of detectors as conventional single-energy CT, which are limited in spatial resolution [8].

Spectral photon-counting computed tomography (SPCCT) has the potential to change the current paradigms [11–13]. This new technology relies on the use of new energy-resolving detectors called photon-counting detectors (PCDs) which bring major improvements compared to EIDs, to the benefit of VMI quality [14]. PCDs are less subject to noise and can be built in a more compact fashion, enabling the use of ultra-high-resolution (UHR) mode, leading to acquisitions with two to three times higher resolution, up to 250 µm. Consequently, spectral images like VMIs and iodine images can be generated in UHR mode [15]. In addition, electrical signals can be separated into different energy ranges known as “bins”, providing a more complete sampling of the transmitted spectrum. Compared to conventional imaging, SPCCT has already shown major improvements in in vitro, as well as in vivo CCTA [15–19].

However, although 70 keV VMIs still serve as the standard image type for CCTA with DECT, SPCCT may offer a new paradigm by providing VMIs of higher quality [20]. However, their impact on CCTA has not yet been thoroughly evaluated. The present study therefore aims to assess the subjective and objective quality of 40 keV SPCCT compared to 70 keV SPCCT, 40 keV DECT, and 70 keV DECT, a high-risk cardiovascular population.

## Materials and methods

### Study design and population

**Fig. 1:**
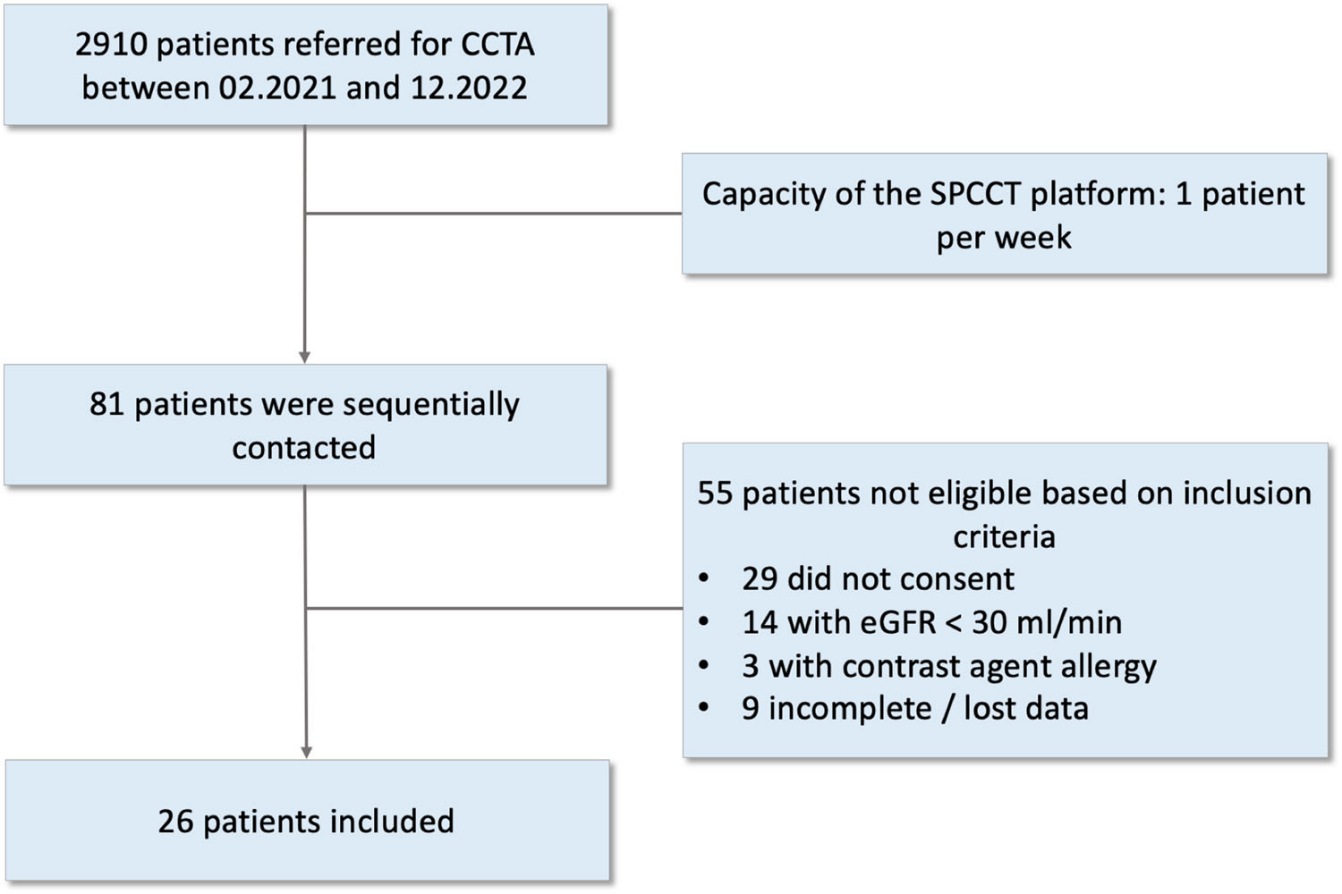
Study flowchart

This was a prospective, comparative, monocentric study conducted at a tertiary cardiothoracic university hospital, (Hôpital Louis Pradel, Hospices Civils de Lyon, France), from February 2021 to December 2022. The local ethics committee approved the study (Hospices Civils de Lyon, approval number: 2019-A02945–52, SPEQUA study). All patients had provided written informed consent. We consecutively included high-risk patients referred for coronary CTA (Fig. 1). CCTA using DECT and SPCCT was performed within 3 days. We reconstructed ultra-high resolution 40 keV and 70 keV SPCCT VMI and compared coronary stenoses with standard resolution 40 keV and 70 keV DECT VMI. All patients < 18-year-old, with a contraindication for iodinated contrast media injection, or renal failure with eGFR < 30 mL/min were excluded.

### DECT and SPCCT systems

Both the DECT and SPCCT systems were manufactured by Philips Healthcare. The DECT systems were the commercially available IQon CT and CT7500, using dual-layer EIDs. The SPCCT system is a clinical prototype with a single-layer energy-sensitive PCD and a large field of view (FOV = 500 mm in plane). Its PCD is made of cadmium zinc telluride with a pixel pitch of 275 × 275 μm^2^ at the isocenter, connected with a proprietary ChromAIX2 application-specific integrated circuit [1]. Further details on the technical aspects of the SPCCT prototype are available in a previously published study [11].

### Acquisition protocol

CCTA was performed with DECT and SPCCT for all patients, within a range of three days. We performed our standard clinical CCTA protocol with both systems, using a retrospective ECG-gating helical acquisition mode with a target heart rate of 60 bpm. Sublingual nitroglycerine (Natispray, Teofarma) and oral beta-blockers (esmolol chlorohydrate [Esmocard, Orpha Devel Handels Vertriebs]) were used when necessary. Depending on the patient’s weight, 65 (< 80 kg) to 75 mL (> 80 kg) of Iomeprol (400 mg/mL; Iomeron®, Bracco) was injected at 5 mL/s, followed by a saline flush. For DECT, a bolus tracking with a region of interest (ROI) in the descending aorta (110 HU threshold) was used, whereas, for SPCCT, we first performed a bolus test of 20 mL of Iomeprol injected at 5 mL/s followed by a saline flush of 20 mL at 4 mL/s.

Images were acquired at 120 kVp and 255 mAs for both systems. No dose modulation was available on the SPCCT. Automatic exposure control was used with both DECT systems, with a DoseRight index of 28 (target current: 255 mAs for an average adult with a water equivalent diameter of 29 cm). CT dose index (CTDI*vol*), as well as dose length product (DLP), was recorded for each patient and compared between DECT and SPCCT.

### Reconstruction parameters

Images were reconstructed at the 78% R–R interval, i.e. during the mid-diastolic phase of the cardiac cycle, with a small FOV of 220 mm and a matrix size of 512 × 512 mm. Voxel size was set at 0.43 × 0.43 × 0.67 mm for both DECT systems, whereas it was set at 0.43 mm^3^ for SPCCT, providing UHR parameters. A medium-smooth XCB (Xres cardiac standard) kernel was used for DECT images and a Detailed 2 kernel was used for SPCCT. Fourth-generation iterative reconstruction algorithms were applied on both systems, with the use of iDose^4^ (Phillips Healthcare), level 3 for DECT and level 6 for SPCCT. Monoenergetic images were generated for each system, at 40 keV and 70 keV (representative of conventional polychromatic images). All acquisition and reconstruction parameters are summarized in Table 1. Attenuation measurements in the aortic root, proximal and distal coronary arteries, along with signal-to-noise ratio (SNR) and contrast-to-noise ratio (CNR) values using epicardial fat as the background, are reported for all reconstructions in Supplemental Table 1.

**Table 1:** Acquisition and reconstruction parameters

	DECT 1 (IQon)	DECT 2 (CT7500)	SPCCT
Acquisition parameters			
Tube voltage, (kVp)	120	120	120
Tube current, (mAs)	255	255	255
Dose modulation	Dose right index of 28 (255 mAs as reference)	Dose right index of 28 (255 mAs as reference)	No modulation
Rotation time, (s/rot)	0.27	0.27	0.33
Pitch	0.16	0.16	0.32
Collimation, (mm)	64 × 0.625	64 × 0.625	64 × 0.275
Focal spot, (mm)	Standard (1.1 × 1.2)	Standard (1.1 × 1.2)	Small (0.6 × 0.7)
Reconstruction parameters			
FOV, (mm)	220	220	220
Matrix size, (pixels)	512 × 512	512 × 512	512 × 512
Slice thickness, (mm)	0.67	0.67	0.25
Reconstruction kernel	XCB (cardiac standard)	XCB (cardiac standard)	Detailed 2
Iterative reconstruction	iDose^4^ 3	iDose^4^ 3	iDose^4^ 6
Denoising algorithm	None	None	SPCNS

*DECT* dual-energy computed tomography, *SPCCT* spectral photon-counting computed tomography, *FOV* field of view, *XCB* Xres cardiac standard

### Qualitative image quality assessment

All images were viewed by two experienced observers with 4 (G.F.) and 9 (S.A.S.-M.) years of experience in cardiac imaging. Images were anonymized and displayed in random order on a clinical workstation (Intellispace Portal, Philips Healthcare). Readers were blinded to patient identity, image origin (DECT vs SPCCT), as well as energy level (40 keV vs 70 keV). Window-level adjustments were allowed according to personal preference. All CCTAs were attributed a subjective quality score from 1 to 5 (1 = unacceptable, 2 = suboptimal, 3 = clinically acceptable, 4 = above average, and 5 = excellent). The criteria under evaluation were: proximal lumen, distal lumen, lumen sharpness, lumen conspicuity, coronary wall, calcified plaque, non-calcified plaque, image noise, overall quality and diagnostic confidence. The presence of artefacts (motion, metallic, and other) was assessed. Coronary segments with strong motion artefacts were excluded from the analysis.

### Quantitative image quality assessment

To quantitatively evaluate image quality, coronary stenoses, when present, were assessed by the same two readers for all patients. The type of plaque (calcified or non-calcified) was noted for each stenosis. Each stenosis was displayed in manual double oblique multiplanar reconstruction, with a cross-sectional and a longitudinal view, and was attributed a visual score (1–24%, 25–49%, 50–69%, 70–99%, and 100% stenosis), following CAD-RADS 2.0 categories [21]. All stenoses with a visual score of 25% or higher on one of the VMIs were quantitatively measured using proximal and distal diameters before and after the stenosis, with the following formula [22]:

$$\frac{{{\rm{Avg.}}}\;{of\; proximal\; and\; distal\; diameters\,-\, Minimal\; lumen\; diameter}}{{{\rm{Avg.}}}\;{of\; proximal\; and\; distal\; diameters}}$$

Values for stenosis measurements were averaged between the two readers.

Blooming artefacts in calcified plaques were evaluated by measuring external and internal diameters according to the following formula [3]:

$${{{\rm{Blooming}}}}=\frac{{{{\rm{External}}}}\; {{{\rm{diameter}}}\,-\,{{{{\rm{Internal}}}}\; diameter}}}{{{{\rm{External}}}}\; {{{\rm{diameter}}}}}\,\times\,100$$

To avoid any inter-reader discrepancies due to manual windowing, window levels were set at the attenuation of coronary artery lumen in a non-calcified area, with a window width equal to 2.5 times the window level [20].

### Statistical analysis

Statistical analysis was performed using RStudio (2023.06.0) [23]. Continuous variables were tested for normality with a Shapiro–Wilk test. Continuous, normally distributed variables are expressed as mean ± standard deviation (SD) and compared with a paired *t*-test. Ordinal, non-normally distributed variables are expressed as a median and interquartile range [IQR] and compared with a Wilcoxon signed-rank test. The proportion of score changes with a 95% confidence interval (CI) was calculated between modalities. Agreement between the two readers for image quality scoring was assessed with the use of a squared weighted Cohen kappa coefficient, defined as: ≤ 0, poor; 0.01–0.20, slight; 0.21–0.40, fair; 0.41–0.60, moderate; 0.61–0.80, substantial; ≥ 0.81, almost perfect (excellent). Coronary stenoses measurements with 40 keV and 70 keV VMI were also compared using a Bland–Altman analysis for SPCCT and DECT with a bias and limits of agreement (LoA) assessment. Statistical significance was defined as *p* < 0.05.

## Results

### Patient inclusion

A total of 26 patients were included (mean age 61 ± 13-years-old, 4 women [15%], BMI 26 ± 6 kg/m^2^) and 36 coronary stenoses were identified. Eight (21%) stenoses were excluded from the analysis due to excessive motion artefacts on SPCCT. There was 1 (4%) stenosis on the left main artery (LM), 16 (57%) on the left anterior descending artery (LAD), 7 (25%) on the circumflex artery (CX) and 4 (14%) on the right coronary artery (RCA). There were 11 (39%) stenoses with calcified plaque and 17 (61%) without. The mean stenosis value, across all reconstructions, was 56% ± 16%. Ninety-six per cent of stenoses were above the 25% threshold (Table 2).

**Table 2:** Population and stenoses characteristics

Parameter	Value
Patients, (*n*)	26 (16%)
Men, (*n*)	22 (84%)
Women	4 (15%)
Age, (yo)	61 ± 13
Weight, (kg)	78 ± 13
BMI, (kg/m^2^)	26 ± 6
Hypertension	11 (41%)
Diabetes	4 (15%)
Hyperlipidemia	8 (31%)
Smoking	12 (46%)
Heart rate, (bpm)	63 ± 10
Coronary stenoses	28
LM	1 (4%)
LAD	16 (57%)
CX	7 (25%)
RCA	4 (14%)
Calcified plaque	11 (39%)
Non-calcified plaque	17 (61%)

If not specified otherwise, data expressed as means ± SD

*BPM* beats per minute, *BMI* body mass index, *LM* left main artery, *LAD* left anterior descending artery, *CX* circumflex artery, *RCA* right coronary artery, *yo* years-old

### Dose analysis

A mean CTDI*vol* of 32 ± 15 mGy was found for DECT and 28 ± 4 mGy for SPCCT, with a statistically significant 6% difference (*p* = 0.02). A mean DLP of 679 ± 387 mGy·cm was found for DECT and 445 ± 53 mGy·cm for SPCCT with a statistically significant 24% difference (*p* = 0.001). Note that CTDI*vol* is more representative of the dose difference between the two modalities as the *Z*-axis covered is shorter with SPCCT.

### Quantitative evaluation

**Fig. 2:**
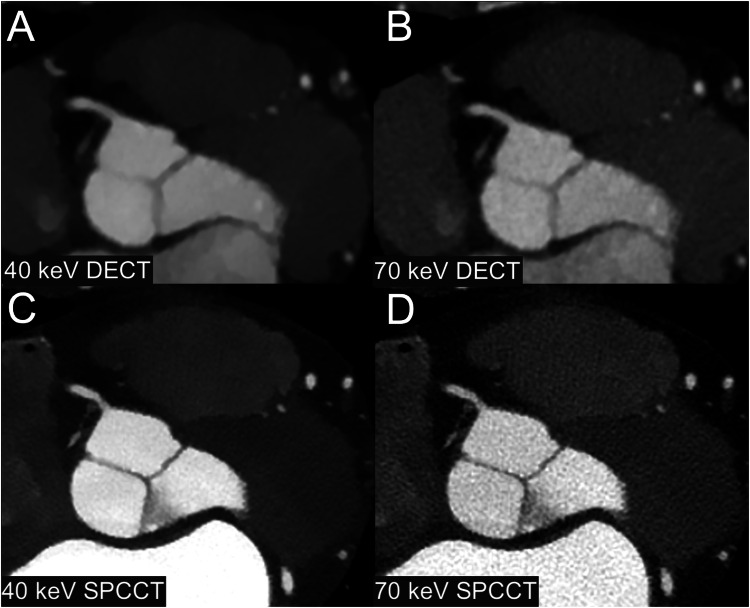
Example of a normal CCTA in a 47-year-old female. **A** 40 keV DECT, (**B**) 70 keV DECT, (**C**) 40 keV SPCCT, (**D**) 70 keV SPCCT

**Fig. 3:**
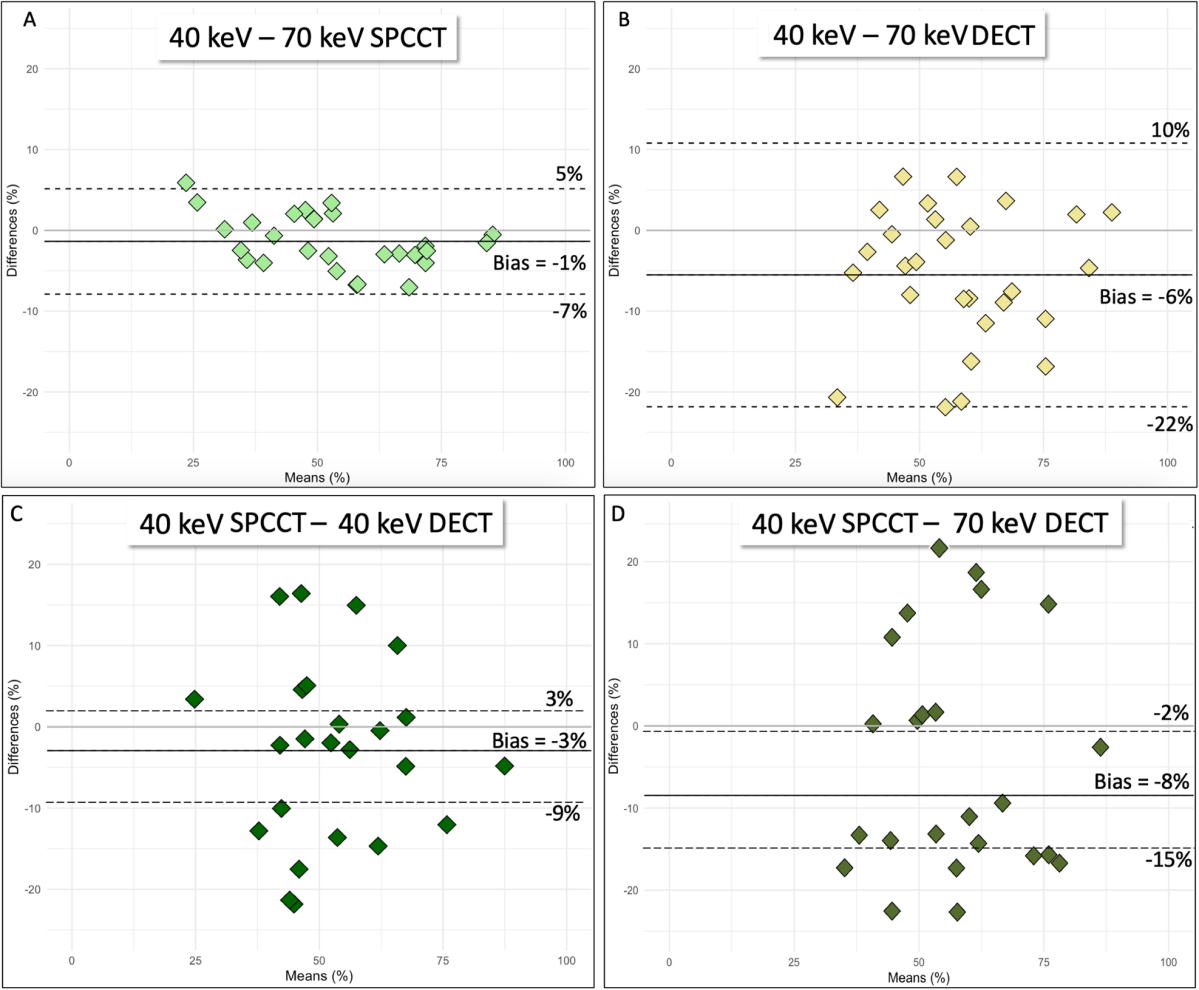
Bland–Altman graphs comparing 40 keV and 70 keV VMIs for SPCCT (**A**) and DECT (**B**), as well as 40 keV SPPCT vs 40 keV DECT (**C**) and 40 keV SPCCT vs 70 keV DECT (**D**)

Examples of normal images are shown in Fig. 2. The mean stenosis value with SPCCT images was 52% ± 16% for 40 keV and 54% ± 17% for 70 keV (*p* = 0.03). The mean stenosis value with DECT images was 55% ± 15% for 40 keV and 61% ± 15% for 70 keV (*p* = 0.015). Bland–Altman analysis showed a greater agreement of stenoses measurements between 40 keV and 70 keV SPCCT (bias: −1% ± 3%, LoA: 6%) than between 40 and 70 keV DECT (bias: −6% ± 8%, LoA 16%) (Fig. 3). Blooming measurements revealed the following means: 40 keV DECT at 59% ± 12%, 70 keV DECT at 52% ± 11%, 40 keV SPCCT at 55% ± 8%, and 70 keV SPCCT at 52% ± 9% (range: 35–66%). The mean blooming difference between 40 keV and 70 keV was 7% ± 6% for DECT and 2% ± 5% for SPCCT. Additionally, the mean blooming difference with 40 keV SPCCT was 1% ± 9% for 40 keV DECT, −1% ± 9% for 70 keV DECT and 2% ± 5% for 70 keV SPCCT. Quantitative quality scores are summarized in Table 3.

**Table 3:** Quantitative stenosis and blooming measurements

Parameter	40 keV DECT, (%)	70 keV DECT, (%)		40 keV SPCCT, (%)		70 keV SPCCT, (%)
Degree of stenosis Mean ± SD [min–max]	51% ± 15% [23–90]	61% ± 15% [39–88]		52% ± 16% [26–85]		54% ± 17% [21–86]
Mean stenosis difference between 40 keV and 70 keV	−6% ± 8%			−1% ± 3%		
Mean stenosis difference with 40 keV SPCCT	1% ± 16%	8% ± 17%				−1% ± 3%
Blooming Mean ± SD [min–max]	59% ± 12% [5–85]	52% ± 11% [39–72]		55% ± 8% [46–71]		52% ± 9% [35–66]
Mean blooming difference between 40 keV and 70 keV	7% ± 6%			2% ± 5%		
Mean blooming difference with 40 keV SPCCT	1% ± 9%	−1% ± 9%				2% ± 5%

Data are in percentages ± SD

*DECT* dual-energy CT, *SPCCT* spectral photon-counting CT

### Qualitative evaluation

**Fig. 4:**
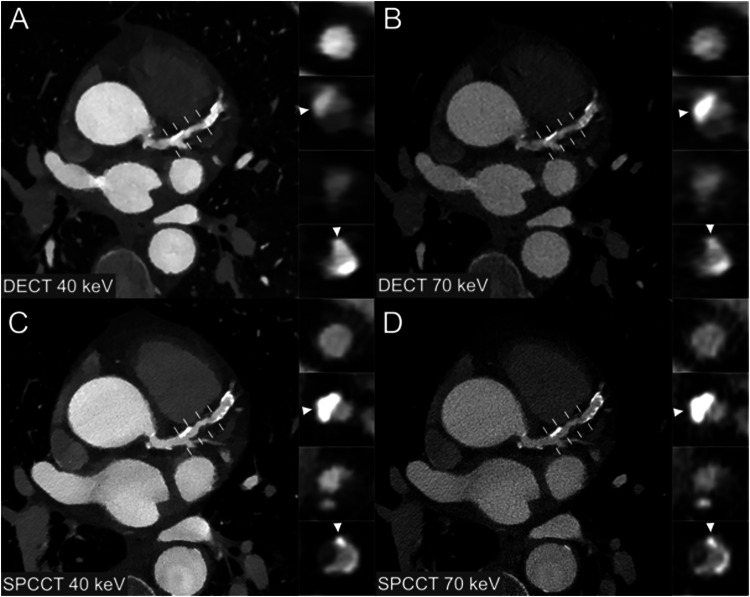
Example of coronary lumen analysis in a 72-year-old female with a complex, mixed-type plaque in the left anterior descending coronary artery. **A** 40 keV DECT, (**B**) 70 keV DECT, (**C**) 40 keV SPCCT, (**D**) 70 keV SPCCT. Enhanced contrast is evident in the 40 keV images compared to those at 70 keV, with SPCCT’s increased resolution enabling improved readability of coronary plaque components

**Fig. 5:**
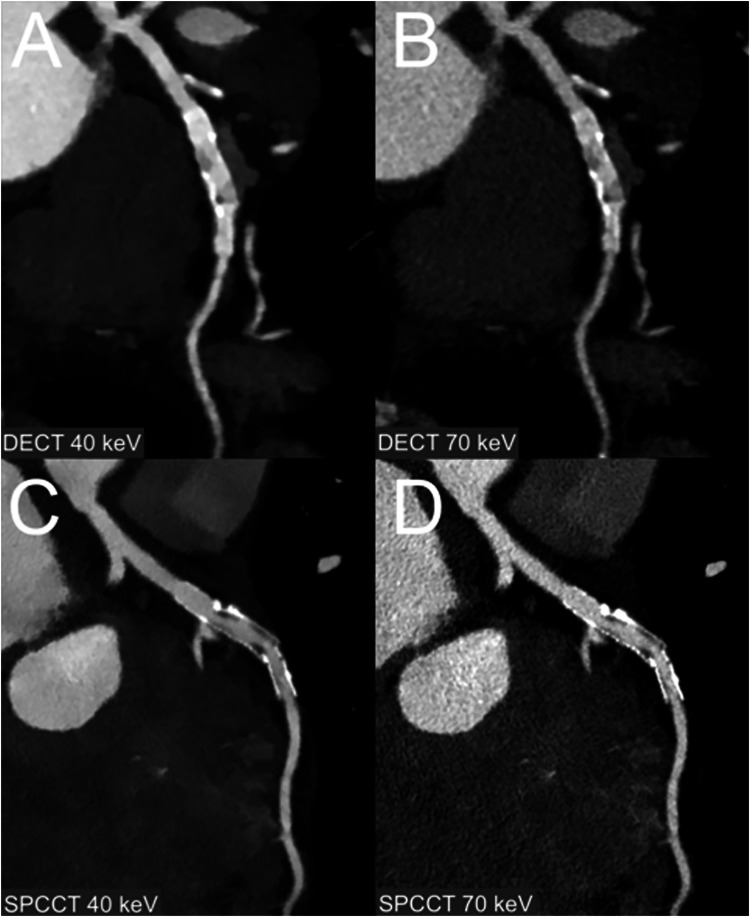
Example of stent analysis in a 67-year-old male with two consecutive stents in the left anterior ascending artery. **A** 40 keV DECT, (**B**) 70 keV DECT, (**C**) 40 keV SPCCT, (**D**) 70 keV SPCCT. Improved stent lumen visibility is noted, distinguishing between intra-stent thrombus and iodine contrast media more effectively, with fewer blooming artefacts on SPCCT, even at 40 keV

**Fig. 6:**
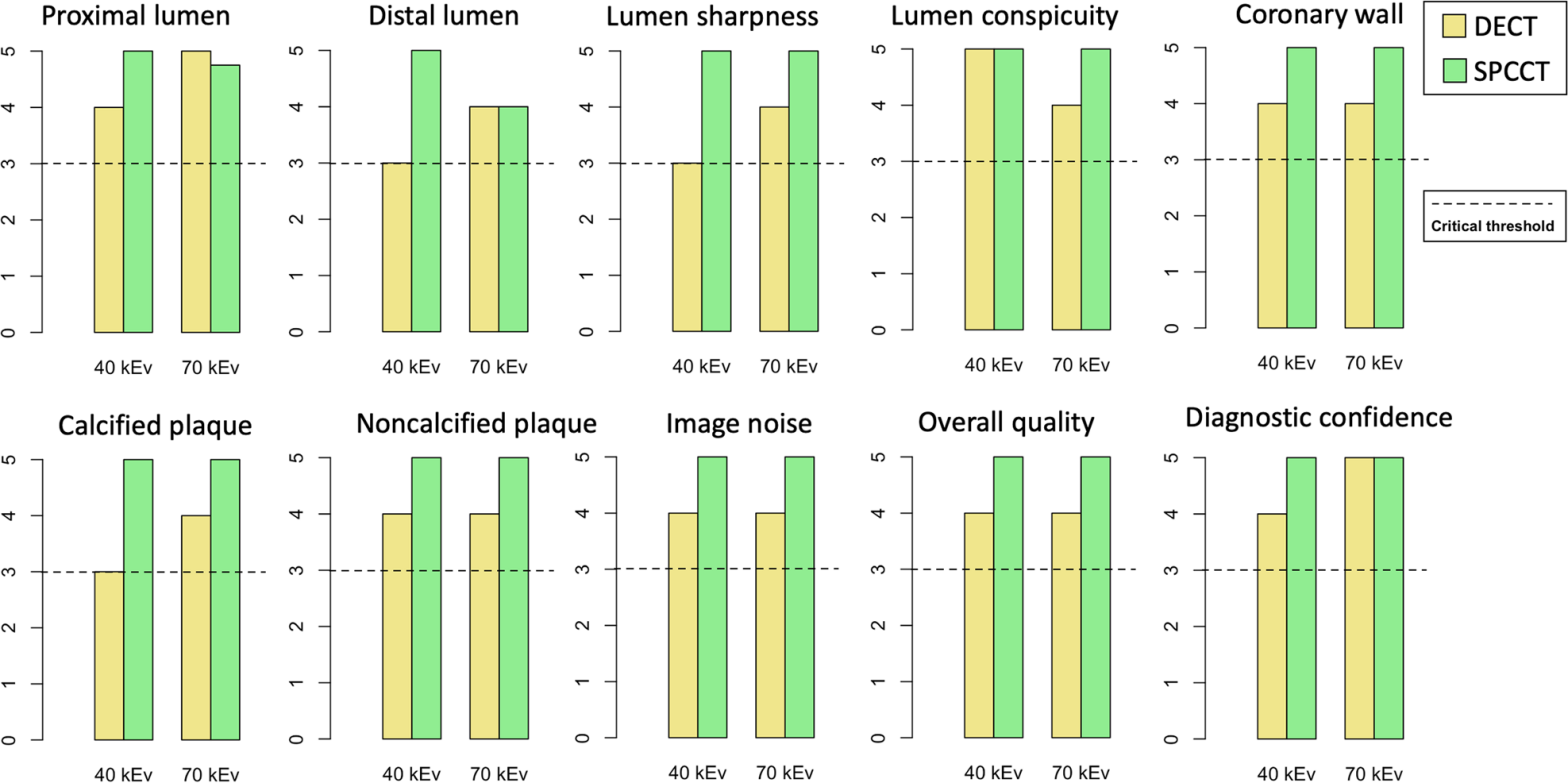
Qualitative analysis comparison of DECT (yellow) and SPCCT (green), separated into 40 keV and 70 keV columns, for multiple image quality parameters

Examples of coronary stenoses and coronary stent visualization with the different keV and modalities are shown in Figs. 4 and 5. 40 keV SPCCT showed a higher overall quality score (5 [5, 5]) compared with 70 keV SPCCT (5 [4, 5], 40 keV DECT (4 [3, 4]) and 70 keV SPCCT (4 [4, 5]), (*p* < 0.001*)*. The biggest improvements were for distal lumen, lumen sharpness and calcified plaque, from median scores of 3 with 40 keV DECT to 5 with 40 keV SPCCT (all *p* < 0.001), with score improvements of 33%, 28%, and 28%, respectively. There were no categories with median scores below 3. Qualitative quality scores are summarized in Table 4 and Fig. 6. Qualitative scores for each reader are summarized in Supplemental Table 2.

**Table 4:** Proportion of quality scores change between modalities, calculated as the percentage improvement or decline from a reference variable to a comparison variable

Parameter	40 keV vs 70 keV all, (%)	40 keV SPCCT vs 70 keV SPCCT, (%)	40 keV SPCCT vs 40 keV DECT, (%)	40 keV SPCCT vs 70 keV DECT, (%)
Proximal lumen	1 [−1, 4]	10 [5, 14]	21 [25, 28]	10 [5, 15]
Distal lumen	−3 [−7, 0]	14 [10, 19]	59 [44, 74]	36 [26, 48]
Lumen sharpness	8 [6, 11]	3 [−1, 6]	50 [39, 62]	17 [10, 24]
Lumen conspicuity	−8 [−11, −5]	5 [0, 10]	1 [−1, 3]	14 [9, 18]
Coronary wall	4 [1, 6]	7 [3, 10]	36 [28, 44]	13 [7, 19]
Calcified plaque	12 [9, 15]	−2 [−4, 0]	41 [35, 47]	15 [9, 22]
Non-calcified plaque	6 [3, 8]	2 [−1, 4]	34 [22, 46]	18 [9, 26]
Image noise	−2 [−5, 1]	3 [0, 5]	21 [16, 26]	20 [11, 22]
Overall quality	6 [3, 8]	1 [−3, 3]	42 [34, 50]	20 [12, 28]
Diagnostic confidence	8 [5, 11]	0 [0, 0]	51 [35, 68]	19 [10, 28]

The reported values include the mean percentage change, with the corresponding 95% confidence intervals

Data are in percentages with [95% CI]

*DECT* dual-energy CT, *SPCCT* spectral photon-counting CT

Excellent inter-reader agreement (kappa = 0.90) was found between the two readers regarding image quality scoring. More specifically, a kappa of 0.87 was found for 40 keV SPCCT, 0.86 for 70 keV SPCCT, while a kappa of 0.86 was found for 40 keV DECT and 0.93 for 70 keV DECT.

## Discussion

In the present study, we demonstrated that ultra-high resolution 40 keV VMIs from SPCCT outperformed both 70 keV VMIs from SPCCT and 40 keV and 70 keV VMIs from DECT for evaluating coronary stenoses. This represents significant progress towards the adoption of spectral imaging as a new standard for CCTA, transitioning from conventional images (or their equivalent 70 keV VMIs) to ultra-high resolution low-keV VMIs with SPCCT. This shift offers numerous advantages regarding image quality and holds the potential for ecological and economic improvements in patient care.

Previous studies have already demonstrated the overall superiority of SPCCT compared with DECT and conventional CT for coronary stenoses [15, 18, 24, 25], as well as coronary stent assessment [19, 26, 27]. Similar results were found in the present study, with an overall better median quality score of 5 for SPCCT vs 4 for DECT. However, fewer studies have tackled the impact of VMIs on coronary assessment. For DECT, 40 keV VMIs led to subjective and objective image quality improvements, but resulted in great variability in quantification compared with 70 keV [7, 28, 29]. This was also observed in the present study, where significant variability in stenosis measurements was found between 40 keV and 70 keV VMIs for DECT, with a 16% LoA. This was associated with poorer overall quality at 40 keV except for lumen conspicuity. On the contrary, UHR 40 keV VMIs yielded less variability in measurements compared with 70 keV VMIs for SPCCT, with a 6% LOA. This was associated with an overall better quality score. This is in agreement with other studies on VMI and SPCCT highlighting improved quality using 40 keV VMIs [14, 17, 30, 31].

One particular concern about 40 keV VMI is its impact on blooming artefacts [32]. Indeed, calcified plaque analysis showed that transitioning from 70 keV to 40 keV resulted in a 7% increase in blooming artefacts with DECT, which could explain part of the variability in measurements and poorer image quality. On the other hand, it only increased by 2% with SPCCT, which may be explained by the benefit of the UHR mode yielding an in-plane resolution of 250 microns [33]. This is also reflected in the qualitative analysis where transitioning from 70 keV to 40 keV resulted in a loss of image quality in 18% of the cases of calcified plaque for DECT but only 2% for SPCCT. This ties in with other studies which have shown a reduction in blooming artefacts with SPCCT using the UHR mode [34, 35], whereas other PCD models without UHR mode have shown persistence of more blooming artefacts in the 40 keV range [36, 37]. Furthermore, it is important to note that although 40 keV VMI with DECT yielded slightly noisier images with a −2% change in image noise score, it was, on the contrary, slightly better with SPCCT, with a + 3% image noise score, which may have been a concern when transitioning from 70 keV to 40 keV.

The present study highlights the great potential for the clinical use of UHR low VMI, possibly leading to additional advantages not yet assessed in this study. First, it may pave the way for low-iodine-volume protocols, thereby lessening the use of contrast media whilst maintaining diagnostic image quality, such as evaluated by multiple studies, not just for coronary arteries [28, 30, 33] but also for other vascular structures such as the aorta [38] or peripheral arteries [39].

The aforementioned advantages of using spectral imaging to improve CCTA protocols and image quality are further enhanced by the introduction of SPCCT. Indeed, it offers higher spatial resolution, greater noise suppression at spatial frequencies, and better lesion detectability than DECT. These improvements, first identified in a phantom setting [14] are now substantiated by the findings of our present study and represent another step towards the promising role that SPCCT may play in CAD imaging. This is also suggested by recent evidence in comprehensive stent imaging, calcium scoring quantification, molecular atherosclerotic plaque characterization and colour K-edge imaging of the coronary lumen [27, 40–46].

However, there are still some technical gaps that need to be filled. Indeed, a high rate of motion artefacts was observed using the current SPCCT prototype system, particularly for the right coronary artery (seven stenoses out of eight excluded), which is expected to have the highest velocity and spatial change compared with other major coronary arteries. This can be explained by the prototype limitations: low coverage, slow rotation, and no motion compensation software. By comparison, no stenoses were excluded using DECT in the present study. Hence, future spectral photon-counting products will have at least the same capabilities as the current DECT systems: a 4–8 cm coverage, a rotation speed of 0.27 s/rotation or more, as well as dedicated software-based solutions. For example, the use of vendor-specific motion-compensated reconstruction for the current generations of dual-layer CTA may reduce motion artefacts by a factor of 11 [47]. These technical limitations are expected to be addressed in future products, predicting further improvements in image quality. Furthermore, statistically significant differences in dose were observed between SPCCT and DECT, with DECT showing higher DLP. However, this parameter is dependent on the *Z*-axis coverage, which is wider for DECT. The smaller difference in CTDI*vol* suggests that these dose variations are unlikely to introduce a significant bias in the results.

### Our study also has certain limitations

First, it is a monocentric study with a limited number of patients included, thus limiting the analysis of subgroups such as CAD-RADS sub-categories or calcified stenoses for blooming assessment. Second, qualitative scores are subjective and may vary between different observers. Third, coronary stenosis was quantified by different CT technologies and image reconstruction techniques but not compared to a gold standard such as invasive coronary angiography. Fourth, the acquisition and reconstruction settings (such as bolus-tracking, cardiac motion correction, resolution, kernel, iterative reconstruction, denoising, motion-compensated reconstruction and radiation dose) vary between scanners. Finally, we used a voxel size of 0.43 mm at the isocenter with a 512-matrix and the Detailed 2 filter which, despite an enhanced spatial resolution, still does not achieve the finest resolutions possible with SPCCT, which opens to further investigations.

## Conclusion

Ultra-high resolution 40 keV SPCCT outperformed 40 keV and 70 keV DECT for assessing coronary artery stenoses, without impairment compared with 70 keV SPCCT, in high-risk patient cohort.

## Supplementary information

ELECTRONIC SUPPLEMENTARY MATERIAL

## Abbreviations

CAD: Coronary artery disease
CCTA: Coronary computed tomography angiography
CT: Computed tomography
DECT: Dual-energy computed tomography
EID: Energy-integrating detector
FOV: Field-of-view
keV: Kilo-electronvolt
LoA: Limits of agreement
PCD: Photon-counting detector
SPCCT: Spectral photon-counting computed tomography
VMIs: Virtual monoenergetic images
