# Supplementary material for: Ultra-high-resolution 40 keV virtual monoenergetic imaging using spectral photon-counting CT in high-risk patients for coronary stenoses
Source: Eur Radiol. 2024 Dec 11;35(6):3042–53. doi: 10.1007/s00330-024-11237-x (PMC12081593; doi:10.1007/s00330-024-11237-x)
Supplement: Supplementary file 1 — ELECTRONIC SUPPLEMENTARY MATERIAL [file 330_2024_11237_MOESM1_ESM.pdf]

Ultra-high-resolution 40 keV virtual monoenergetic imaging using spectral photon-counting CT in high-risk patients for coronary stenoses

ELECTRONIC SUPPLEMENTARY MATERIAL

Supplemental Table 1. Attenuation measurements in the aortic root, proximal and distal coronary arteries, along with signal-to-noise ratio (SNR) and contrast-to-noise ratio (CNR) values using epicardial fat as the background, for all reconstructions. SNR is defined as the attenuation divided by the noise. CNR is defined as the attenuation difference divided by the square root of the sum of the squared noise values.

**Parameter**	**40 keV DECT**	**70 keV DECT**	**40 keV SPPCT**	**70 keV SPCCT**
Aortic root attenuation (HU) ± sd	1519 ± 30	457 ± 22	1355 ± 51	443 ± 44
Aortic root SNR	56	23	29	11
Aortic root CNR	45	20	23	10
Proximal coronary artery attenuation (HU) ± sd	1658 ± 86	409 ± 13	1205 ± 75	402 ± 54
Proximal coronary artery SNR	30	13	20	8
Proximal coronary artery CNR	27	13	16	8
Distal coronary artery attenuation (HU) ± sd	906 ± 81	314 ± 38	1205 ± 84	354 ± 55
Distal coronary artery SNR	20	13	22	8
Distal coronary artery CNR	18	12	15	8
Epicardial fat attenuation (HU)	-165 ± 25	-93 ± 21	-54 ± 43	-86 ± 41

DECT, dual-energy CT; SPCCT, spectral photon-counting CT.

Supplemental Table 2. Median and [IQR] quality scores for the two readers

Parameter	40 keV DECT	70 keV DECT	40 keV SPPCT	70 keV SPCCT
Reader 1				
Proximal lumen	4 [4, 5]	5 [4, 5]	5 [5, 5]	4.5 [4, 5]
Distal lumen	4 [3, 4]	4 [3, 4]	5 [4.25, 5]	4 [4, 4]
Lumen sharpness	4 [4, 5]	4 [4, 5]	5 [4.25, 5]	5 [4, 5]
Lumen conspicuity	5 [5, 5]	4 [4, 5]	5 [5, 5]	5 [5, 5]
Coronary wall	4 [3, 4]	4 [4, 5]	5 [5, 5]	5 [4, 5]
Calcified plaque	3 [3,4]	4 [4, 5]	5 [4.5, 5]	5 [5, 5]
Non-calcified plaque	4 [3, 4]	5 [4.5, 5]	5 [4.5, 5]	5 [4, 5]
Image noise	4 [4, 4]	4 [4, 4]	5 [5, 5]	5 [4, 5]
Overall quality	4 [3, 4]	4 [4, 5]	5 [5, 5]	5 [4.25, 5]
Diagnostic confidence	4 [3, 4.75]	5 [4, 5]	5 [5, 5]	5 [5, 5]
Reader 2				
Proximal lumen	4 [4, 5]	4.5 [4, 5]	5 [4.25, 5]	5 [4, 5]
Distal lumen	3.5 [2.25, 4]	5 [5, 5]	5 [5, 5]	4 [4, 4]
Lumen sharpness	3 [3, 4]	4 [3.25, 5]	5 [4.25, 5]	5 [4, 5]
Lumen conspicuity	5 [5, 5]	4 [4, 5]	5 [5, 5]	5 [5, 5]
Coronary wall	4 [3, 4]	4 [3.25, 5]	5 [5, 5]	5 [4, 5]
Calcified plaque	3 [3, 4]	4 [4, 5]	5 [4.5 5]	5 [5, 5]
Non-calcified plaque	4 [4, 5]	5 [4., 5]	5 [4.5, 5]	5 [4.5, 5]
Image noise	4 [3.25, 4]	4 [4, 4]	5 [5, 5]	5 [4, 5]
Overall quality	4 [3, 4]	4 [4, 5]	5 [5, 5]	5 [4.25, 5]
Diagnostic confidence	4 [3, 4]	5 [4, 5]	5 [5, 5]	5 [5, 5]
DECT, dual-energy CT; SPCCT, spectral photon-counting CT. Data are in percentages with [95% CI]				
